# Immuno-informatics Analysis to Identify Novel Vaccine Candidates and Design of a Multi-Epitope Based Vaccine Candidate Against *Theileria* parasites

**DOI:** 10.3389/fimmu.2018.02213

**Published:** 2018-10-15

**Authors:** Prajna Parimita Kar, Anand Srivastava

**Affiliations:** Laboratory of Molecular Interactions, National Institute of Animal Biotechnology, Hyderabad, India

**Keywords:** *Theileria*, immuno-informatics, multi-epitope vaccine, subunit vaccine, TLR4, MHC-I, B-cell epitope, T-cell epitope

## Abstract

Theileriosis poses a serious threat to ruminants in tropical and subtropical countries. It is a tick-borne disease, caused by an apicomplexan parasite, *Theileria*. The high disease burden in animals causes huge economic losses to marginal farmers. Further, with increasing cases of resistance to commonly used drugs, it is highly desirable to develop better and cost-effective vaccines against theileriosis. The only available vaccine, live attenuated parasite vaccine, has many drawbacks and hence is unsuitable for controlling this disease. Immuno-informatics has emerged as a useful tool in down selection of potential molecules for vaccine development. In this study, we have used an immuno-informatics driven genome-wide screening strategy to identify potential vaccine targets containing important and effective dominant immunogens against *Theileria*. The proteome of *Theileria annulata* was screened for proteins with probability of plasma membrane localization or GPI anchor. The proteins non-homologous to the host (bovine) were selected and their antigenicity was analyzed. The B-cell epitopes were identified in the selected proteins and mapped in the modeled structure of the proteins. A total of 19 linear epitopes in 12 proteins, exposed in the extracellular space and having the potential to induce protective antibodies were obtained. Additionally, CTL epitopes which are peptides with 9-mer core sequence, were also identified, modeled and docked with bovine MHC-I structures. The CTL epitopes showing high binding energy with the bovine MHC-I were further engineered *in silico* to design a putative multi-epitope vaccine candidate against *Theileria* parasites. The docking studies and molecular dynamics studies with the predicted multi-epitope vaccine candidate and modeled bovine TLR4 exhibited strong binding energy, suggesting that the complex is stable and the putative multi-epitope vaccine candidate can be a potentially good candidate for vaccine development.

## Introduction

*Theileria* spp. are tick-borne protozoan parasites which belong to the subphylum apicomplexa which includes a number of other important pathogens such as *Plasmodium, Babesia, Toxoplasma, Cryptosporidium*, and *Eimeria* species. *Theileria* spp. are predominantly parasites of ruminants and are transmitted transstadially to the host by ticks (1, 2). These parasites cause huge economic loss to farmers in tropical and subtropical countries. Among various species of these parasites, the most important ones include *T. parva* and *T. annulata*, which cause economically important diseases in cattle, east coast fever and tropical theileriosis, respectively. *T. parva* is transmitted by *Rhipicephalus appendiculatus* whereas *T. annulata* is transmitted by several species of *Hyalomma*.

The disease, theileriosis, is characterized by the lymphoproliferation of the host leucocytes after invasion by the parasites (3). Other symptoms include fever, lymph node enlargement and anemia. The untreated animal usually dies within 3–4 weeks of the infection. This disease kills over million animals every year and significantly reduces the productivity of the cattle (4, 5). The current control measures for theileriosis include the use of acaricides (for controlling vector), chemotherapy (drug such as buparavaquone) and vaccination. Traditionally, control of tick infection has been performed by the application of acaricides. However, it is effective only when performed at the community level and regularly for a prolonged period. The indiscriminate use of acaricides has been leading to resistance in tick, thus adding another dimension to the problem. The chemotherapy agents such as parvaquone, buparvaquone, and halofuginone are effective during the initial stages of infection (6). However, these chemotherapy agents are quite expensive and are not a good choice for the marginal farmers. In addition, reports of development of resistance to these chemotherapy agents are a matter of deep concern (7). The only available vaccine for theileriosis is a live attenuated vaccine (8). However, this vaccine needs a cold chain for transportation and storage which leads to a huge increase in its cost (approximately $8–12 per dose). Further, some vaccinated cattle, especially immunocompromised cattle, remain as a source of transmission of parasites to other cattle (9).

The subunit/multi-epitope based vaccines are generally considered to be safer as compared to live attenuated vaccines (10). Also, the cost of production and distribution of subunit/multi-epitope vaccine is comparatively low, hence these would be economical for the marginal farmers. Thus, developing a subunit and/or multi-epitope vaccine for theileriosis is highly desirable. However, the efforts to develop subunit vaccines against *T. annulata* and *T. parva* have not been successful till date (11).

Long-lasting immunity against *Theileria* can be developed in cattle by repeated challenge with *T. parva* and *T. annulata* sporozoites, by developing neutralizing antibodies (12). These neutralizing antibodies recognize the p67 protein of *T. parva* (13). Immunization with recombinant p67 antigen has been shown to induce immunity in 50% of vaccinated cattle (14). Also, antibodies against parasite protein SPAG1 and Tams1 in *T. annulata* blocks the invasion of leucocytes by sporozoite (15–17).

A large body of evidence indicates that cellular immunity also plays a major role in protection against *T. annulata* and *T. parva*. The immunity can be adoptively transferred by CD8+, but not CD4+ T cells from vaccinated animals. This strongly suggests that cytotoxic T lymphocytes (CTL) play an important role in developing immunity against *Theileria* parasites (18, 19). Further, major histocompatibility complex (MHC) class I-restricted CD8+ cells, the cytotoxic lymphocytes (CTLs), that target schizont-infected lymphocytes also play a primary role in mediating immunity against *Theileria* in cattle (20, 21).

The availability of comprehensive genomic, transcriptomic and proteomic datasets of *Theileria* parasite have provided opportunities for *in silico* mining of novel candidates for vaccine design (22–24). Immuno-informatics, which integrates the transcriptomics and proteomics through advances in computational and molecular immunological tools, has emerged as a new tool for identification of the target antigens for vaccine development (25–27). Since the experimental methods are difficult and time-consuming, the immuno-informatics approach can narrow down a vast number of potential molecules to be tested, thus increasing the chance of finding better candidates. Numerous studies have shown that epitopes based vaccines could be effective in elucidating protective immunity against various pathogens such as influenza A, hepatitis B and C virus, *Leishmania* and *Shigella* (28–32). In this study, using immuno-informatics-driven vaccine target screening strategy, the available transcriptomic and proteomic data for *Theileria* parasites were analyzed to predict proteins/epitopes for vaccine development that could elicit protective humoral and cellular immune response. For an effective humoral response, the antigen must be antigenic and should possess B-cell epitopes, and for effective CTL response, the peptide must be presented by MHC-I to the T cell receptor. Thus, the model antigens for developing subunit/multi-epitope vaccine against *Theileria* should either possess B-cell epitopes or be displayed by MHC-I to CTLs. Using immuno-informatics, we have identified 12 proteins containing 19 epitopes which are likely to elicit the humoral immune response. We have further designed a multi-epitope subunit vaccine by combining CTL epitopes which have the potential to elicit the cellular immune response against *Theileria* parasites (*T. annulata* and *T. parva)*. The results provide a strategy for epitope-based vaccine development for *Theileria* parasites and indicate that immuno-informatics could be an alternative strategy to rapidly discover potential antigens for subunit/multi-epitope vaccine development against parasites.

## Materials and methods

**Figure 1:**
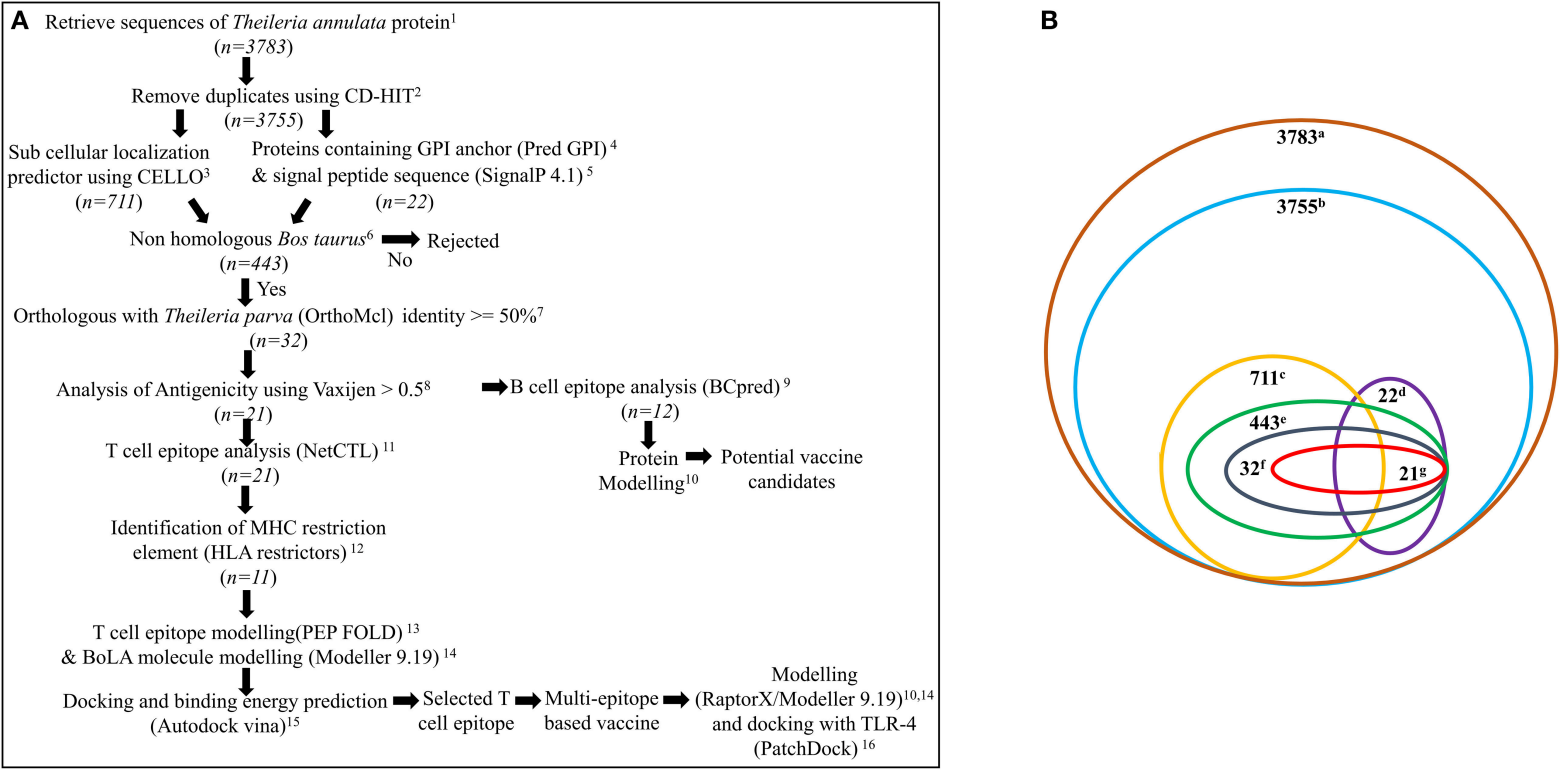
**(A)** Systematic workflow for identification of potential vaccine candidates and construction of multi-epitope subunit vaccine against *Theileria* parasites from the available proteomic data. n represents total number of proteins used for analysis. ^1^https://www.ncbi.nlm.nih.gov/; ^2^http://weizhong-lab.ucsd.edu/cdhit-web-server/cgi-bin/index.cgi?cmd=cd-hit; ^3^http://cello.life.nctu.edu.tw/; ^4^http://gpcr.biocomp.unibo.it/predgpi/pred.htm; ^5^http://www.cbs.dtu.dk/services/SignalP/; ^6^ https://blast.ncbi.nlm.nih.gov/Blast.cgi?PAGE=Proteins; ^7^http://orthomcl.org/orthomcl/; ^8^http://www.ddg-pharmfac.net/vaxijen/VaxiJen/VaxiJen.html; ^9^http://ailab.ist.psu.edu/bcpred/; ^10^ http://raptorx.uchicago.edu/ ^11^http://www.cbs.dtu.dk/services/NetCTL/; ^12^http://www.cbs.dtu.dk/services/HLArestrictor/; ^13^http://mobyle.rpbs.univ-paris-diderot.fr/cgi-bin/portal.py#forms::PEP-FOLD3; ^14^Modeller9.19; ^15^AutodockVina; ^16^https://bioinfo3d.cs.tau.ac.il/PatchDock/; **(B)** Number of proteins obtained after various analysis. a, total proteins of *T*. *annulata*; b, proteins after removal of duplicates; c, plasma membrane proteins; d, proteins containing GPI anchor & signal peptide; e, proteins which are non-homologous to bovine; f, orthologous proteins specifically present in *T. annulata* and *T. parva*, g. antigenic proteins.

A systematic workflow (Figure 1A) was generated for the identification of potential vaccine candidates and construction of multi-epitope subunit vaccine against *Theileria* parasites (*T. annulata* and *T. parva*) from the available transcriptomic/proteomic data (22–24).

### Sequence retrieval and sub-cellular localization

All protein sequences of *T. annulata* were retrieved from the National Centre for Biotechnology Information (NCBI) database. The CD-HIT server was used to remove proteins having 90% or more similarity. All the parameters recommended by the server were set to default (33). The CELLO v.2.5 server was used for the identification of subcellular localization of all the selected proteins. The plasma membrane proteins were selected with a confidence score of ≥2 (34). Further, all the proteins were analyzed for the presence of signal peptide sequence and GPI anchor using SignalP 4.1 and PredGPI server respectively, with default settings (35, 36).

### Screening proteins non-homologous to the host and orthologous to *T. parva*

The selected plasma membrane proteins and proteins having both the signal sequence and GPI anchor were further analyzed for screening the proteins which are non-homologous to the host, *Bos taurus*, using BLASTp server (37) with a cut-off *E*-value 0.005. Proteins predicted with *E*-value more than 0.005 were likely to be non-homologous to *Bos taurus* and selected for further analysis. Further, an orthologous search between *T. annulata* and *T. parva* was conducted. The query strategy was designed in such a way that the average identity between two proteins would be greater than 50%, and would be specific only to *T. annulata* and *T. parva*.

### Antigenicity of proteins

The selected proteins (non-homologous to *Bos taurus* and orthologous to *T. parva*) from the previous step were analyzed for their immunogenicity using Vaxijen - v2 (38). The vaxijen is based on an alignment-free approach for antigen prediction using auto cross-covariance (ACC) transformation of protein sequences into uniform vectors of principal amino acid properties. The classification by Vaxijen is solely based on the physicochemical properties of proteins. The threshold was kept to 0.5 to predict probable antigenic and non-antigenic proteins.

### B-cell epitopes prediction

The prediction of B-cell epitopes for all the selected antigenic proteins from the previous step was performed using BCPREDS server with 90% specificity (39). BCPREDS server (B-cell epitope prediction) is based on Support Vector Machine (SVM) classifiers and the training dataset containing 701 linear B-cell epitopes from Bcipep database and 701 random non-B-cell epitopes from SwissProt sequence database.

Further, the proteins were analyzed for the presence of putative transmembrane domains, exposed and intracellular region(s) using TMHMM server (http://www.cbs.dtu.dk/services/TMHMM/) (40). Finally, those epitopes which were exposed to the extracellular surface of the cells were selected for further analysis.

### Model building, refinement and stereochemistry analysis of proteins predicted with B-cell epitopes

The tertiary structure of the selected proteins which possess B-cell epitopes was built using RaptorX which is a web portal for protein structure and function prediction (41). RaptorX uses a threading-based modeling approach for model building. RaptorX server predicts three-dimensional structures for protein sequences without close homologs in the Protein Data Bank (PDB). RaptorX server can predict secondary and tertiary structures, contacts solvent accessibility, disordered regions and binding sites. It can also assign some confidence scores to indicate the quality of a predicted 3D model such as *p*-value for the relative global quality, GDT (global distance test) and uGDT (un-normalized GDT) for the absolute global quality, and modeling error at each residue. The selected epitopes were then mapped on the structure using the information obtained from the TMHMM prediction.

### Prediction of T cell epitope(s) and their specific MHC-I restriction element(s)

The prediction of cytotoxic T-cell (CTL) epitopes was done using NetCTL 1.2 server (42). The threshold was set to 0.98 for high sensitivity and accuracy. All antigenic proteins were analyzed for the presence of CTL epitopes. The CTL epitopes identified from the previous step were further screened for bovine MHC–I restriction element identification using HLArestrictor 1.1 (43). All 77 bovine MHC-I alleles present in the database were analyzed for their ability to bind to identified CTL epitopes and their binding affinity for respective MHC-I allele was also calculated. The threshold for strong binder (IC_50_) was set to 50 and percentile rank was set to 0.5. The threshold for weak binder (IC_50_) was set to 500 and percentile rank was set to 2.

### Model building, refinement, and stereochemistry analysis of bovine MHC-I

The sequences of bovine MHC-I alleles, namely BoLA-3^*^05101, BoLA-1^*^00901, and BoLA-1^*^00902 were downloaded from the Immuno Polymorphism Database (IPD) (44). Since crystal structures for these proteins were not present, a reliable model was built using homology modeling. The best template for the homology modeling was obtained through protein BLAST (pBLAST) against Protein Data Bank (PDB) and the three-dimensional model of BoLA-MHC proteins were built using modeler 9.19 (45). The generated model was then refined by ModRefiner (46) and stereochemistry analysis was done using PROCHECK, a protein parameters analysis tool (47).

### Tertiary model prediction for CTL epitopes

PEP-FOLD server was used to predict the three-dimensional structure of all CTL epitopes (48). Five probable structures were predicted by this server. The structure having the lowest energy model was selected as the final model for the respective epitope.

### Interaction studies between selected MHC-I allele and CTL epitope

The strong binder pairs of MHC-I allele and CTL epitope obtained from HLA-restrictors were then used for molecular docking studies using AutoDock vina (49). The hydrogen atoms were added to the selected MHC-I alleles and the file was then converted to pdbqt format for docking studies with the CTL epitopes. For BoLA-3^*^05101, grid was set to −23.025, −15.521, −28.935 for X, Y, and Z co-ordinates, respectively. The grid box for BoLA-1^*^00901 was set to 60.708, 61.642, 26.373 for X, Y, and Z co-ordinates, respectively. In the case of BoLA-1^*^00902 the grid box for X, Y, and Z was set to 58.68, 64.412, 33.956 co-ordinates, respectively. The binding affinity of the ligands with their receptors was measured in kcal/mol.

### Building and characterization of the multi-epitope subunit vaccine

All CTL epitopes selected after MHC-I restriction element analysis and interaction studies were fused together with the help of a linker AAY to build a multi-epitope vaccine molecule. The multi-epitope vaccine molecule thus obtained was analyzed for its non-homology to bovine (as described earlier). The antigenic properties of the multi-epitope vaccine candidate was estimated by Vaxijen server (as described earlier). The allergenicity of the vaccine candidate was evaluated using AlgPred (http://crdd.osdd.net/raghava/algpred/) (50). The physiochemical properties such as amino acid composition, theoretical pI, instability index etc., of the vaccine candidates were calculated by ProtParam (http://web.expasy.org/protparam/) (51).

### Structure characterization of the multi-epitope vaccine candidate and bovine TLR4

A template-based tertiary structure prediction of the multi-epitope vaccine candidate was carried out by RaptorX server (41). The further refinement of modeled multi-epitope vaccine candidate was done using ModRefiner server as described previously (46) and stereochemistry analysis was done using PROCHECK (47).

The sequence of bovine TLR4 was downloaded from UniProt. Since crystal structure for this protein was not present, a reliable model was built using homology modeling. The best template for homology modeling was obtained through BLASTp against PDB database. A three-dimensional model of bovine TLR4 protein was built on modeler 9.19 using full-length human TLR4 as a template. The generated model was then refined by using ModRefiner and stereochemistry analysis was done using PROCHECK.

### Molecular docking of multi-epitope vaccine candidate with bovine TLR4 receptor

The interaction studies between bovine TLR4 and final subunit vaccine protein were carried out with the help of a protein-protein docking server PatchDock (52). The results were refined according to their binding score by FireDock server (53).

### Molecular dynamics simulation of bovine TLR4 receptor with multi-epitope vaccine candidate

To determine the stability of a protein-ligand complex molecular dynamics (MD) simulation study of the complex is an important tool. Molecular dynamics simulation was carried out for the docked complex of the TLR4 and multi-epitope vaccine protein for 100 ns with a time interval of 2fs using Maestreo software. The simulation was done with Single Point Charge (SPC) water as a solvent model and 0.15 M NaCl buffer. The boundary conditions were set to orthorhombic (a=b=c=10 Angstrom; α = β = γ = 90°). The isothermal-isobaric pressure (NPT) ensemble were set at 300 K (temperature) and 1 atm pressure. Nose-Hoover Thermostat method was used to maintain the temperature constant. To examine the standard deviation and fluctuation of the backbone of the protein, the root mean square deviation (RMSD) and root mean square fluctuation (RMSF) analysis were performed.

## Results

### Identification of plasma membrane and GPI anchored proteins of *Theileria* parasite

A systematic workflow based on the immuno-informatics approach was designed to identify the potential vaccine candidates which would elicit an effective antibody response and to design a multi-epitope based vaccine candidate which would generate effective cell-mediated immune response against *Theileria* parasites (Figure 1A). The first step in this workflow was to screen for *T. annulata* proteins having the potential to be localized on the cell surface. The complete transcriptome/proteome of *T. annulata* comprising total 3,783 protein sequences was downloaded from NCBI database for this purpose (Figure 1B). The paralogous sequences with more than 90% similarity were identified using CD-HIT server from all the protein sequences. Twenty-eight protein sequences were removed as paralogs while remaining 3755 protein sequences were selected for further analysis. The selected 3755 protein sequences were submitted to the CELLO sub-cellular localization server to screen for the proteins predicted to be localized on the plasma membrane. The output of this software is in the form of different numerical values as confidence scores for predicting the probability of localization of the proteins in the cell. A total of 711 proteins obtained with the confidence score greater than 2 were selected for further analysis (Figure 1B).

Furthermore, the criteria chosen for identification of proteins predicted to be localized on the cell surface was that it should have a signal peptide along with a GPI anchor. All the 3755 protein sequences were screened for the presence of a signal peptide sequence and a GPI-anchor. Only 390 out of the 3755 proteins were found to possess a signal peptide sequence based on SignalP 4.1 analysis. From PredGPI analysis for GPI anchors, only 49 proteins containing GPI-anchors were obtained. Only 24 out of these 3755 proteins were predicted to contain a signal peptide sequence as well as a GPI-anchor (Figure 1B).

Ten out of these 24 proteins were also present in the plasma membrane analysis. After adding the results from CELLO for proteins predicted to be localized at the plasma membrane, and proteins having both the signal peptide sequence and the GPI-anchor signal sequence, 726 protein sequences were finally selected for further analysis (Figure 1B).

### Identification of antigenic *T. annulata* proteins non-homologous to *Bos taurus* and orthologous to *T. parva*

All 726 protein sequences from previous analysis based on the cell surface localization were analyzed for their non-homology to the host (*Bos taurus*) and orthology to *T. parva*. BLASTp analysis against *Bos taurus* resulted in 443 proteins with *E*-value above 0.005. It is assumed that these proteins are not present in the host (Figure 1B).

The OrthoMCL server was used for searching the orthologous or conserved protein between *T. annulata* and *T. parva*. Out of the 443 selected proteins non-homologous to *Bos taurus*, only 32 proteins were found to be conserved and present exclusively in *T. annulata* and *T. parva*. These 32 proteins were selected for further screening (Figure 1B).

All 32 protein sequences selected from the previous step were submitted to Vaxijen web server to predict the antigenic properties of each protein. Vaxijen web server is an efficient tool for predicting antigenic properties of proteins with an accuracy of 70–89%. Out of the 32 protein sequences, only 21 protein sequences were predicted as probable potent antigens with the threshold of 0.5 (Tables S1, S2). Fifteen out of 21 protein sequences were found to be hypothetical.

### Potential proteins of *Theileria* parasites for peptide/subunit vaccine development

**Figure 2:**
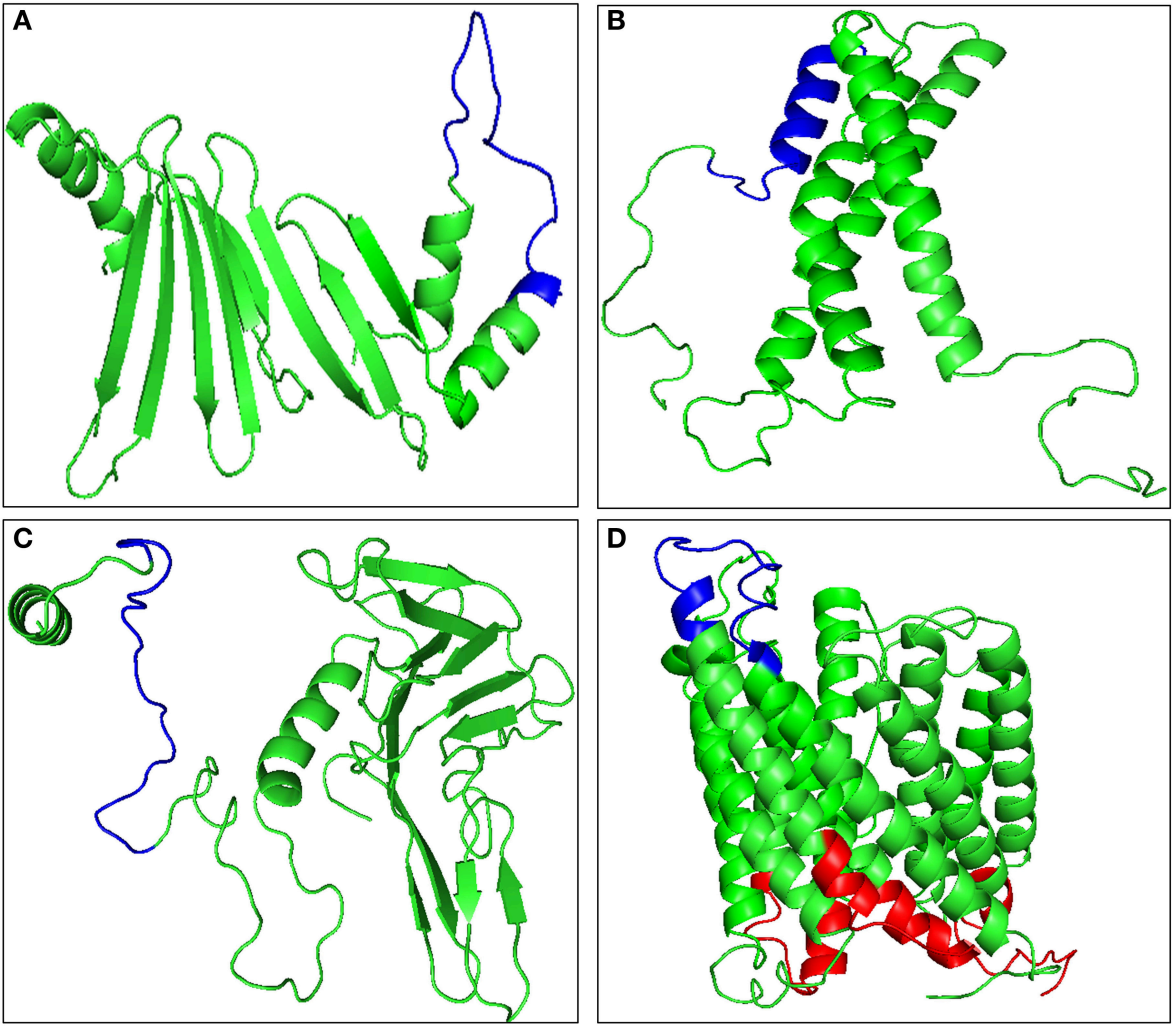
Molecular modeling of antigenic proteins of *Theileria annulata* containing B-cell epitope using Raptor X. **(A)** TA09755, **(B)** TA13065, **(C)** TA13810, and **(D)** TA13385. Identified epitopes mapped onto the modeled proteins are shown in red color for buried epitopes, and blue color for exposed epitopes using TMHMM.

B-cell epitopes are the antigenic determinant regions which are recognized by the specific antibodies. The predicted antigenic proteins were then analyzed for 20 amino acid long linear B-cell epitopes. BCPREDS server was used to predict the probable B-cell epitope and the specificity was kept at 90% or more to minimize chances of false positive. Four proteins, namely TA15480, TA21140, TA09756, and TA13665, were predicted not to possess any B-cell epitope with the defined cut off value (Table S3). The B-cell epitope must be exposed to the extracellular regions of the cell to be recognized by the antibodies. Hence the remaining 17 proteins with putative transmembrane domains were analyzed for the exposed and buried regions using TMHMM server (Table S4). The tertiary structures of all these proteins were built using RaptorX and the exposed epitopes were mapped on the structure (Figure 2, Figure S1). Five proteins, namely TA15965, TA05820, TA14950, TA15795, and TA12045, were found to have no exposed B-cell epitopes, while the remaining 12 proteins contained B-cell epitopes exposed to the cell surface (Figure 2, Figure S2). A total of 19 B-cell epitopes were predicted in these 12 proteins (Table 1). Presence of merozoite-piroplasm surface antigen (TA17050) which is one of the leading vaccine candidates for the subunit vaccine against *T. annulata* (54) in our final list of 12 proteins validates the *in silico* analysis. TA17050 contains five B-cell epitopes with good peptide score (more than 0.97 for all peptides) and an antigenic score of 0.5708. The remaining 11 proteins also are potentially good targets for the development of subunit vaccine, especially TA09755 (FLPQTSRPSMGKKKGSFQLP-with peptide score 0.976) exhibited the highest antigenic score (0.7931). Tpr-related protein family member (TA13385), which is unique to the *Theileria* parasite, could also be considered as a good target for developing a subunit vaccine. Eight other peptides from the list with scores more than 0.99, could be evaluated as potential peptide-based vaccine candidates (Table 1).

**Table 1:** Antigenic proteins with their peptide and antigenic score.

**S. No**.	**Gene ID**	**Position**	**Peptide sequence**	**Peptide score**	**Antigenic score**
1	TA09755	132	FLPQTSRPSMGKKKGSFQLP	0.976	0.7931
2	TA13065	157	KKPASDFEEQALEEYLKDKD	0.964	0.7595
3	TA13810	193	DEKEETSKKKYVLMVVVVVV	0.972	0.7115
4	TA13385	336	AVGFPSITENWDSTAATGNG	0.993	0.6889
		375	EYKRHDPSKWPTDGMTTRTA	0.985	
5	TA16735	912	VNWEYVWEKEYKYYRMKMSN	0.986	0.6443
6	TA17050	16	VISSVNAANEDEKKKEEKKD	1	0.5708
		105	NLHPAQPKMFKKKGDKEYSE	1	
		212	FYTGDSRLKETYFELKDDKW	0.994	
		243	LNAMNSSWSTDYKPVVDKFS	0.991	
		43	TSCENVTFKNVDSNTTELTV	0.978	
7	TA11900	198	ELTTTKLVNVIVNGTQESIN	0.984	0.578
8	TA13820	24	EKNEQVTIDINKDATNPRKN	0.985	0.5741
9	TA17055	175	HTVERDDESEEEAAITRVCC	0.999	0.5708
		39	LGYTLDTTIITSIGRDKINR	0.986	
10	TA16125	99	NVTEGGTSMYKYFVKVKGKW	0.963	0.5466
11	TA21100	32	YNSTQNDTNTPRTGSYYNAV	0.999	0.5342
12	TA12115	251	FINFICKIIPPGFPGQLLIQ	1	0.5083
		37	LVKKTLFNRETDPSNNNFPF	0.963	

### Identification of cytotoxic T-cell epitopes specific to bovine MHC-I restriction elements

The predicted 21 antigenic proteins were analyzed for the CTL epitopes using NetCTL server. A total of 179 CTL epitopes were predicted with a threshold value of 0.98 (Table S5). The CTL epitopes work in conjugation with the MHC-I present on the surface of antigen presenting cells. Hence, using HLA-restrictors server, all the 179 CTL epitopes were analyzed for their ability to be recognized by MHC-I restriction elements of *Bos taurus* and the binding affinity of each CTL epitope with bovine MHC-I allele was predicted. Only 11 proteins containing 27 epitopes were predicted to have a strong binding affinity (IC_50_ value <50 and percentile rank below 0.5) with MHC-I molecules (Table S6). Twenty-two out of these 27 predicated T-cell epitopes were predicted to have a strong binding affinity with BoLA-3^*^05101, 14 out of these 27 epitopes were predicted to have a strong binding affinity with BoLA-1^*^00902, and 9 out of these 27 epitopes were predicted to have a strong binding affinity with BoLA-1^*^00901. Although many more MHC-I molecules were obtained which were predicted to have a strong binding to selected CTLs, but only these 3 MHC-I molecules (BoLA-3^*^05101, BoLA-1^*^00902, and BoLA-1^*^00901) were predicted to have a strong binding affinity for multiple epitopes (Table S6).

### Molecular modeling and structure refinement of bovine MHC-I allele

The tertiary structure of BoLA-3^*^05101 was predicted by Modeler 9.19 with bovine MHC-I molecule N^*^01801 (BoLA-A11) (3PWU) as the template. BoLA-3^*^05101 showed 84% identity with N^*^01801. The root mean square deviation (RMSD) between the structure of modeled BoLA-3^*^05101 and structure obtained after refinement was found to be 0.489 Å. The predicted tertiary structure of BoLA-3^*^05101 contained 47.64% coils, 21.05% helices and 31.30% beta strands. The Ramachandran plot of this modeled protein obtained by PROCHECK suggested that this was a highly reliable model (Figure S2).

Both BoLA-1^*^00902 and BoLA-1^*^00901 showed 76 and 86% identity, respectively with cattle MHC-I N^*^01301 (2XFX'A') presenting an 11mer peptide from *T. parva* chain A. The RMSD value of modeled BoLA-1^*^00902 and BoLA-1^*^00901 protein, before and after refinement, was calculated to be 2.682 and 1.713 Å respectively. The predicted structure of BoLA-1^*^00902 contained 48.74% coils, 23.4% helices, and 27.86% beta strands while the predicted structure of BoLA-1^*^00901 was found to have 45.54% coils, 24.02% helices and 30.44% beta strands. The Ramachandran plot of modeled proteins obtained by PROCHECK showed that the modeled proteins were stable (Figure S2).

### Modeled bovine MHC-I interacts strongly with identified CTL epitopes

**Figure 3:**
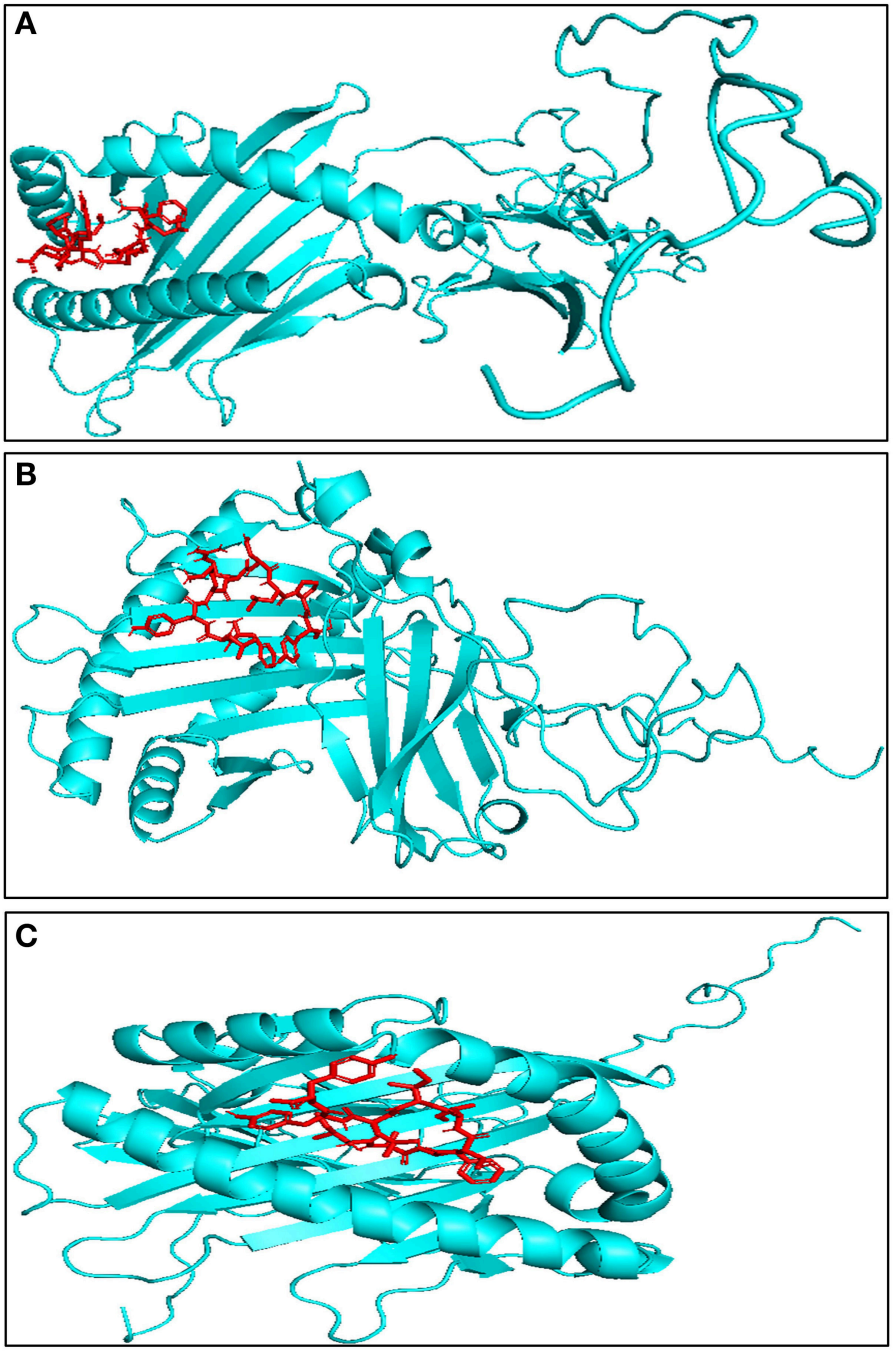
Representative model of interaction between predicted CTL epitope through molecular docking studies **(A)** FLYKRDLPY with BoLA-N:05101, binding energy = −7.8 kcal/mol, **(B)** FLYKRDLPY with BoLA-N:00902, binding energy = −7.7 kcal/mol, **(C)** WMVFFIVVY with BoLA-N:00901, binding energy = −7.6 kcal/mol. Red represents CTL epitope, and blue represents modeled bovine MHC-I.

It is necessary for the CTL epitopes to interact with the respective MHC-I molecule with high binding affinity for generating specific immune response. In order to find the affinity between bovine MHC-I allele and identified CTL epitope, molecular docking studies were performed using AutoDock Vina. All 27 CTL epitopes modeled using PepFold server were docked with their respective MHC allele. The predicted CTL epitopes “FVAWFYKLY” and “FLYKRDLPY” exhibited the highest binding affinity of−7.4 kcal/mol while “ILFTISLHY” exhibited the lowest binding affinity of −5.5 kcal/mol with BoLA-N: 05101 MHC allele (Figure 3, Table 2). The predicted CTL epitope “FLYKRDLPY” showed highest binding affinity of −6.7 kcal/mol and “IAFCIILYY” showed the lowest binding affinity of −5.1 kcal/mol with BoLA-N: 00901 MHC allele. The predicted CTL epitopes “IAFCIILYY” displayed the highest binding affinity of −7.2 kcal/mol and “STIAMGLVY” displayed the lowest binding affinity of −4.4 kcal/mol with BoLA-N: 00902 MHC allele.

**Table 2:** Interaction studies of modeled bovine MHC-I with CTL epitopes.

**MHC-I**	**CTL epitopes**	**Binding energy (Kcal/mol)**
**Interaction studies with BoLA-N:05101 and CTL epitopes**		
BoLA-N:05101	LYEYVLYLY	−5.8
	FVAWFYKLY	−7.4
	FSNLYSGYY	−6.7
	FLYKRDLPY	−7.4
	IAFCIILYY	−6.9
	FINLTLLTY	−6.4
	ITDVLIYIY	−6.8
	ILLTFNHLY	−7.1
	ILFTISLHY	−5.5
	CSLYFVVLY	−6.3
	KMGHLTIYY	−6.4
	STIAMGLVY	−6.5
	SSFNILLSY	−6.4
	FINLVHYYY	−6.2
	FTEHNSLEY	−6.6
	YSLLFFYLY	−5.8
	SLLFFYLYY	−6.9
	FALDIMTKY	−5.7
	YIKEYFSLY	−7.1
	VVFDYSVKY	−5.5
	VFSIVSSLY	−6.5
	FFELLPSLY	−6.7
**Interaction studies with BoLA-N:00901 and CTL epitopes**		
BoLA-N:00901	LVLVGSLSY	−5.6
	FLYKRDLPY	−6.7
	WMVFFIVVY	−6.4
	IAFCIILYY	−5.1
	KMGHLTIYY	−6.6
	STIAMGLVY	−5.6
	SSFNILLSY	−6.6
	YSLLFFYLY	−6.3
	VVFDYSVKY	−5.6
**Interaction studies with BoLA-N:00902 and CTL epitopes**		
BoLA-N:00902	LVLVGSLSY	−6
	FLYKRDLPY	−6.8
	WMVFFIVVY	−6.1
	IAFCIILYY	−7.2
	ILLTFNHLY	−4.8
	KLVNLILLY	−5.6
	MVIAVLSHY	−6.1
	ILFTISLHY	−6.3
	KMGHLTIYY	−6
	STIAMGLVY	−4.4
	SSFNILLSY	−5.6
	YSLLFFYLY	−5.9
	LLVNHTYHY	−5.5
	VVFDYSVKY	−6.5

Further, TA12045 contains the highest number of CTL epitopes which showed strong affinity to 18 bovine MHC-I. Another protein, TA16125, contains 4 CTL epitopes which showed strong affinity to 12 bovine MHC-I (Table S6). Thus these proteins, namely TA15965, TA12045, and TA16125, can also be considered as good candidates for T cell-mediated response against *Theileria* parasites.

### Design of multi-epitope subunit vaccine and prediction of allergenicity, physiochemical and antigenic properties

Based on the predicted binding affinity score of CTL epitope and bovine MHC-I restriction element analysis, all the 27 CTL epitopes were fused together with a linker “AAY” to form the multi-epitope subunit vaccine molecule. The final vaccine was composed of 321 amino acid residues. The allergenicity of the vaccine construct was predicted using AlgPred against BLAST search on allergen representative peptides (ARPs). The result showed that the multi-epitope vaccine candidate would not act as an allergen. The molecular weight and theoretical pI of this multi-epitope subunit vaccine candidate were calculated as 3.79 and 6.75 kDa respectively. Based on the theoretical pI calculation this vaccine candidate was found to be slightly acidic in nature. Further, the antigenicity of this potential vaccine candidate was predicted to be 0.7286 as analyzed by Vaxijen server, suggesting that it would be antigenic in nature.

### Homology modeling and model refinement of bovine TLR4 and multi-epitope vaccine candidate

**Figure 4:**
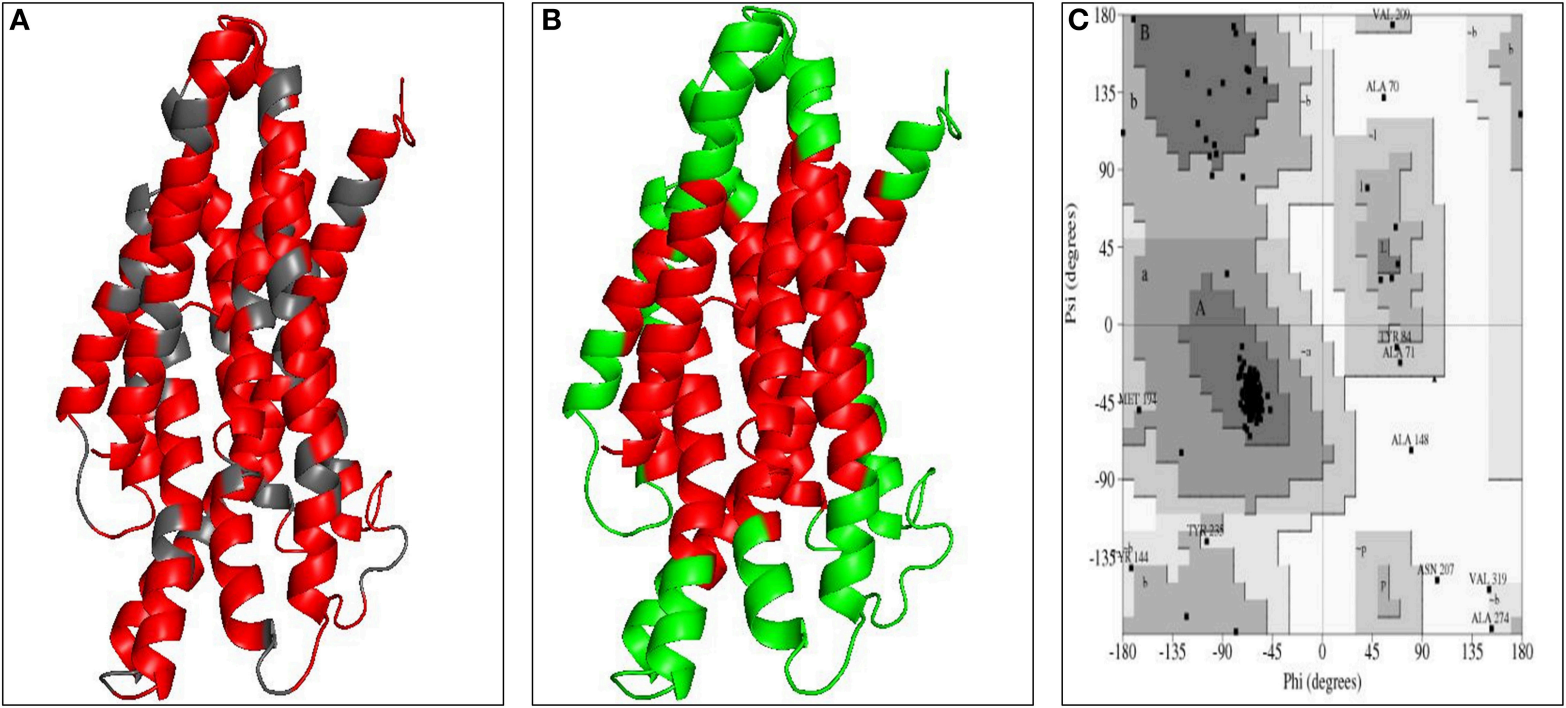
Modeled structure of multi-epitope based subunit vaccine candidate. **(A)** Three dimensional model of multi-epitope based subunit vaccine candidate obtained by homologous modeling and refinement. The red color represents CTL epitopes and gray color represents AAY linker, **(B)** B-cell epitopes of multi-epitope based subunit vaccine candidate represented in green color, **(C)** Ramachandran plot analysis of the multi-epitope based subunit vaccine candidate showing favored (91.7%), allowed (4.8%), generously allowed (2.6%), and disallowed (1%) regions.

The three dimensional model of the multi-epitope vaccine candidate was generated using the RaptorX server as no template was found in the PDB database. The RaptorX server predicted only a single domain for this multi-epitope vaccine candidate. The best template for the multi-epitope vaccine candidate was found to be 4AV3'A' with its *p*-value 6.61e^−05^ (Figure 4A). The Ramachandran plot of this protein revealed that 89.1% amino acids were located in the core region, 8.3% amino acids were located in the allowed region, 1.6% amino acids were located in the generously allowed region and none was located in the disallowed region (Figure 4C). The predicted secondary structure of the multi-epitope vaccine candidate was found to possess only helices (Figure S3A).

**Figure 5:**
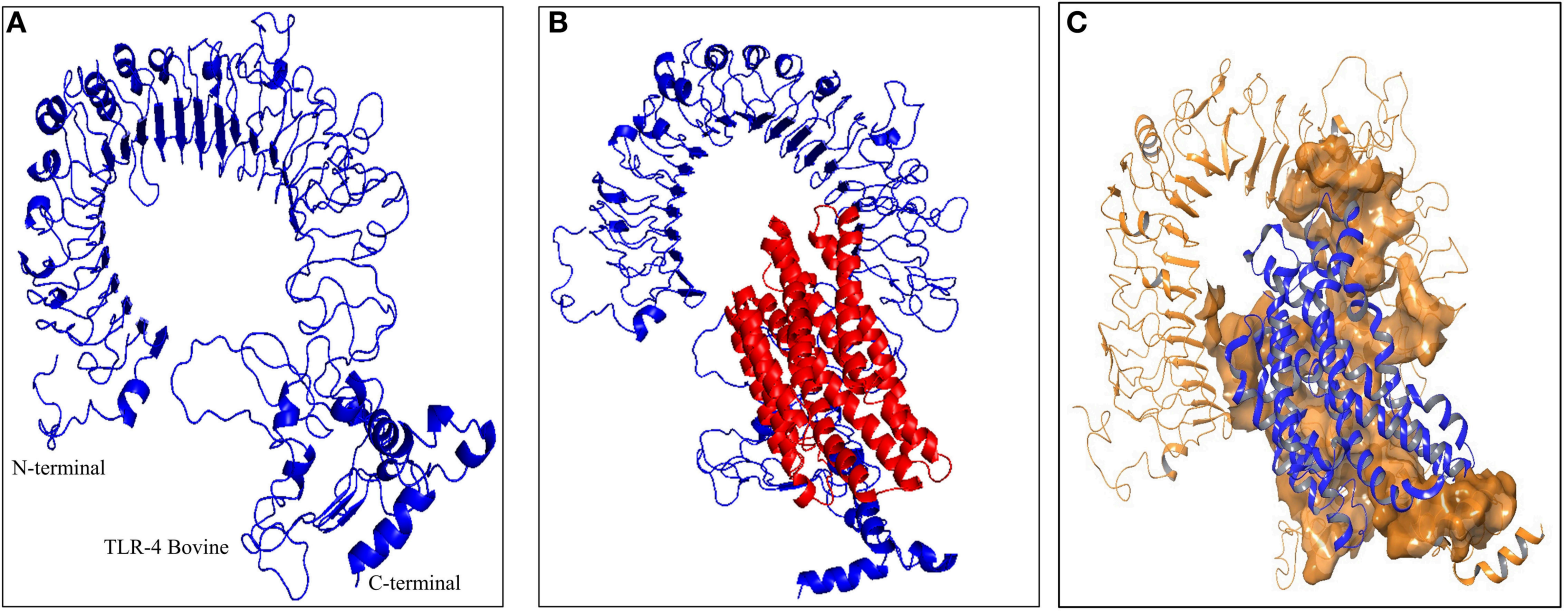
Docking studies of multi-epitope vaccine candidate and modeled bovine TLR4. **(A)** Three dimensional model of bovine TLR4 obtained by homologous modeling and refinement, **(B)** Docked complex of TLR4 and multi-epitope based subunit vaccine candidate, **(C)** Binding sites of multi-epitope vaccine candidate with bovine TLR4. The interacting residues of the putative multi-epitope vaccine candidate and bovine TLR4 are presented as surface model.

As the crystal structure of bovine TLR4 was not available, the tertiary structure of bovine TLR4 was modeled using 4G8A (human TLR4) as a template by Modeler 9.19 software (Figure 5A). Both the query coverage and identity were found to be 72%. The structure was then refined using ModRefiner and validated with PROCHECK analysis. The Ramachandran plot of the modeled bovine TLR4 revealed that 79.6% amino acids were located in the core region, 17.4% amino acids were located in the allowed region, 1.8% amino acids were located in the generously allowed region and only 1.2% amino acids were located in the disallowed region.

### Multi-epitope vaccine candidate possesses CTL and B-cell epitopes specific to *Theileria* parasites but not to the bovine host

The analysis for the presence of CTL epitopes in the multi-epitope vaccine protein by the NetCTL server showed the presence of a total of 49 CTL epitopes. Out of the 49 epitopes, 26 epitopes were found to be the same as the previously predicted epitopes in our analysis (Table S7). Remaining 23 epitopes were the newly predicted CTL epitopes. All the newly predicted CTL epitopes were subjected to BLASTp against bovine for non-homology search and it was found that none of these epitopes were present in the bovine. Further, the discontinuous B-cell epitopes were also predicted from the structure of the multi-epitope vaccine protein using EliPro server (55). A total of 9 conformational B-cell epitopes were predicted by the server (Figure 4B, Table S8, Figure S3B). Similar to the CTL epitopes, all the predicted B-cell epitopes were also analyzed for their non-homology to the bovine and it was found that none of these B-cell epitopes were present in the bovine proteome database. These results suggest that this potential multi-epitope vaccine protein is non-homologous to bovine and does not possess any predicted B-cell and CTL epitopes from bovine. Hence, the putative multi-epitope vaccine candidate would not generate an autoimmunity or tolerance in bovine.

### Molecular docking of the multi-epitope vaccine candidate with bovine TLR4 receptor

To predict the binding energy of this multi-epitope vaccine candidate with the bovine TLR4, docking of this multi-epitope vaccine candidate with TLR4 was carried out using PatchDock server based on the shape complementarity principle (Figure 5B). PatchDock server generated 20 complexes which were scored according to the protein surface, geometry and electrostatic complementarity. The top 10 complexes were then submitted to the FireDock server in order to refine and re-score the docking solutions of TLR4 and multi-epitope vaccine candidate. The final docking model was chosen from the molecular docking studies based on the binding score. The best structure of the multi-epitope vaccine candidate and bovine TLR4 showed a binding energy of −18.3 kcal/mol (Figure 5B). The attractive van der Waals force was calculated as −36.97 kcal/mol, the repulsive van der Waals force was 49.96 kcal/mol, and the atomic contact energy was −2.10.

### Molecular dynamics simulation of TLR4 receptor with multi-epitope vaccine

**Figure 6:**
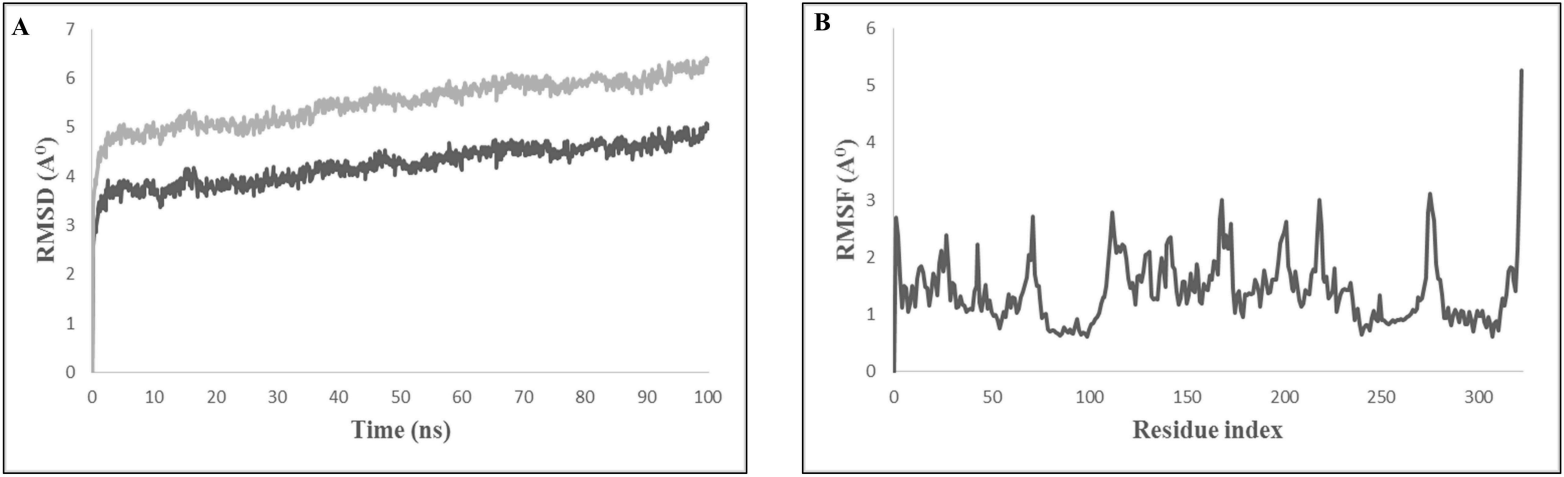
Molecular dynamics simulation study of TLR4 and multi epitope vaccine protein complex. **(A)** Root Mean Square Deviation (RMSD) with a time duration of 100 ns, gray line represents side-chain and black line represents backbone of the protein chain **(B)** Root mean Square Fluctuation (RMSF) of the docked complex side-chain with a time duration of 100 ns.

The evaluation of the stability of the bovine TLR4 and multi-epitope vaccine candidate complex using molecular dynamics showed that the complex was quite stable in SPC water type with 0.15 M NaCl. The root mean square deviation (RMSD) of the backbone of the protein and the root mean square fluctuation (RMSF) for all the side chain amino acid residues were analyzed for a time period of 100 ns. The interaction between the TLR4 and the multi-epitope vaccine protein was found to be stable after 10 ns (Figure 6A). The RMSF of side chain residues was found to be in between 1 and 3Å with a little variation (Figure 6B). A total of 42 hydrogen bonds were formed between the TLR4 receptor and the multi-epitope vaccine protein. The simulation analysis clearly shows that the multi-epitope vaccine protein can be recognized by the TLR4 and form a stable complex.

## Discussion

Various factors contributing to the failure of any antigen as a vaccine candidate under development stages are: (a) antigen not being exposed to immune response (b) poor antigenic/immunogenicity response of antigen and, allergenicity to the host. Recently, with the advancement of *in silico* analysis it has been possible to identify molecule(s) which would likely possess maximum qualities/properties to qualify as a good antigen. The primary aim of this study was to identify the candidate gene(s) having the potential to induce an immune response against macroschizont stage of *T. annulata*. We utilized a systematic immuno-informatics approach in this study to predict potential vaccine candidate(s) in *Theileria* parasites using the proteome databases of *T. annulata* and *T. parva*, to design a multi-epitope based vaccine.

One important criteria for selecting a potential antigen for vaccine development is that it should be located on the surface of the cell so that it is more accessible for both the humoral and the cellular immune system. The proteins are translocated to the cell surface using a signal sequence for plasma membrane translocation or could be anchored to the Glycosylphosphatidylinositol (GPI) moieties. These proteins which are exposed to the extracellular surface are easily recognized and are likely to elicit an immune response when used as a target antigen for vaccine development. Proteins from the *Theileria* proteome database were thus examined for their translocation to plasma membrane using CELLO software. CELLO is based on support vector machine (SVM) system to predict the probability distribution for possible localization of the protein which is more reliable than the other sub-cellular localization prediction tool, PSORTb. Furthermore, PSORTb program is trained only for the bacterial proteome (56). We selected a confidence score of 2 or more for the sub-cellular localization prediction based on our observation that in many cases the values lesser than 2 resulted in predicting two different sub-cellular localizations for the same protein. A total of 711 proteins were predicted to be localized at the cell surface using the CELLO prediction server. To take into account the proteins that may also be present on the cell surface using GPI moieties, we first looked for the proteins having signal peptide sequences using the SignalP 4.1 server. Few research groups have also analyzed the proteome of *T. annulata* and *T. parva* for the identification of proteins containing signal peptide sequence and GPI anchor using the older version of SignalP (23, 57–59). However, number of false positives in SignalP 4.1 is lesser compared to the older versions (*http://www.cbs.dtu.dk/services/SignalP/performance.php*). SignalP 4.1 uses a Hidden Markov Model (HMM) method for predicting the presence of signal peptide in the protein. However, it does not predict the GPI anchors unlike the previous versions. PredGPI server was used to identify the GPI anchored proteins in our study. The PredGPI uses a prediction method which is coupled to HMM and SVM methods. PredGPI has been shown to outperform the other methods used for predicting GPI-anchors as it has a lower rate of false positive predictions with respect to the other available methods (35). Only 22 proteins were predicted to possess both the signal peptide and GPI anchor. The putative plasma membrane and the GPI anchored proteins obtained from both the analysis were added for further analysis.

In order to generate an immune response, the antigen must be recognized as non-self. Hence, the antigen for which homologous protein is present in the host is not considered a good candidate for vaccine development. Thus, we removed all the proteins homologous to *Bos taurus* in our study. Only 443 out of the 733 proteins were predicted as non-homologous to *Bos taurus*. Further, an ideal vaccine candidate should be capable of stimulating an effective immune response against various species of *Theileria*. Thus, to increase the stringency, the query was set in such a way that only *T. annulata* or *T. parva* proteins would be screened.

Antigenicity is one of the important features of an antigen for vaccine development. Vaxijen is the only server which uses alignment-independent prediction of protective antigens. It classifies antigen based on the physiochemical properties of the proteins. The prediction efficiency of this method is found to be 70–80%. We finally found 21 proteins which were predicted as antigenic and also qualified other screening conditions.

An effective vaccine should induce a specific immune response against specific pathogens/antigen by selectively stimulating antigen-specific B-cells or CTLs or T helper cells. In order to initiate the humoral response, antigens must be exposed and should be recognized by the naive B-cells. Further, to initiate the cellular response, naive T cells need to be primed by the peptide antigens bound to the major histocompatibility complex (MHC) molecules on the antigen presenting cells (APCs). The activation of CTLs (CD8+ cells) is a result of the specific engagement of a 9 mer peptide to the MHC-I proteins. The CTLs eliminate the cells which they recognize as non-self, hence the immune system must be able to discriminate between healthy and infected cells.

Thus, the B-cell epitopes were predicted using the BCPREDS server. Further, B-cell epitopes must be accessible to the immune response. Hence all the proteins containing B-cell epitopes were modeled and the epitopes mapped on the structure. Four proteins did not show any B-cell epitopes and epitopes on 5 other proteins were not accessible to immune recognition. Finally, 11 proteins were predicted to be good targets for the development of subunit vaccine. One of the 11 proteins was TA17050 which is one of the leading vaccine candidates, thus suggesting the likeliness of the other 10 proteins as good candidates for subunit vaccine development.

The CTL epitopes in all the antigenic proteins were identified using NetCTL server. NetCTL is a quite reliable tool for the prediction of cytotoxic T lymphocytes and the predictions done by this are based on proteasomal cleavage, transporter associated with antigen presentation (TAP) transport efficiency and MHC class I affinity. In comparison to the earlier version NetCTL1.0, the accuracy of prediction of cytotoxic T lymphocytes using NetCTL server is significantly improved (42). Although NetCTL is a tool designed for the prediction of human CTL epitopes in a given protein, it can be used for bovine as well with equal efficiency. A total of 179 CTL epitopes were identified using this prediction method. Previously, studies conducted to identify CTL epitopes from *T. parva* specific CD8+ T cell line by transfecting cells with parasite cDNA identified 10 *T. parva* antigens having a total of 15 CTL epitopes (60). It was initially intriguing to observe that none of the antigens identified as CTL epitopes by this group were present in our analysis. However, after careful analysis, we found that the 10 proteins that this study identified did not possess either a signal sequence and GPI anchor together, or they were not predicted to be localized to the plasma membrane. Using a similar strategy, three proteins (TA15705, TA17545, and TA14970) were shown to be recognized by CD8 T cells from *T. annulata* infected cattle (61). TA14970 neither contains a signal sequence nor a GPI anchor. Although TA15705 (Ta9) and TA17545 possess the signal sequence, but these proteins lack a GPI anchor. Hence, we did not include these proteins in our further analysis. The CTL epitopes must be presented by MHC-I on the surface of the antigen presenting cells to the T-cell receptor for the activation of cell-mediated response. Hence, we predicted the binding energy of these CTL epitopes with all the bovine MHC-I molecules using HLA restrictor server. The binding pairs which showed the highest energy were selected for further analysis. Three MHC-I molecules, namely BoLA-3^*^05101, BoLA-1^*^00902, and BoLA-1^*^00901, were found to be the best, both in terms of binding energy and recognition to multiple CTL epitopes. Hence these three molecules were further modeled to predict their three-dimensional structures. Protein models having more than 90% of the residues in the core and allowed regions are generally considered as high-quality models. We found that the predicted model for BoLA-3^*^05101, BoLA-1^*^00902, and BoLA-1^*^00901 were high-quality models based upon the presence of more than 90% of their amino acid residues in the core and allowed regions. The docking of selected CTL epitopes with modeled BoLA-3^*^05101, BoLA-1^*^00902, and BoLA-1^*^00901 further confirmed the stability of the complex. Thus, 27 CTL epitopes were found to be potential peptides for generating effective CTL response. Immunization with all these twenty-seven, 9 mer peptides for the generation of CTL response would be a tedious process. Hence we designed a new protein containing all the CTL epitopes joined with AAY linker. Immunization with a single protein (approximately 3.79 kDa) would be much easier and cost effective if it could generate an effective cellular response. Thus, we re-analyzed our newly designed multi-epitope vaccine candidate for its ability to serve as an ideal vaccine candidate. The newly designed molecule was predicted to be non-allergic while it was antigenic in nature. The multi-epitope vaccine candidate was found to possess 49 CTL epitopes and none of them were found to be present in the bovine, and 26 were specifically from *Theileria* parasites.

Since the multi-epitope vaccine candidate is a novel protein, hence no template was found for homologous modeling. Accordingly, RaptorX was used for generating a model for this protein. The possibility of the generated model being worse than the best of a set of randomly generated models can be calculated by *p*-value. A lesser *p*-value represents the higher quality model. For the alpha helix protein *p*-value should be lesser than 10^−3^, and for the beta-sheet protein *p*-value should be lesser than 10^−4^. The predicted structure of the multi-epitope subunit vaccine contained all alpha helices with a *p*-value of 6.61e^−05^ which indicate that the structure predicted by RaptorX is a high-quality model. The un-normalized Global Distance Test (uGDT) also estimate the model quality, like, if a protein contains greater than 100 residues and if the uGDT score is more than 50, it is a good indicator for high quality model. The final multi-epitope vaccine candidate had the uGDT score of 73. Forty-four percent of the protein residues were exposed in the subunit vaccine protein, while it is generally assumed that if 15% of the protein residues are exposed and solvent accessible, they can generate effective humoral and cellular immune response.

The modeled tertiary structure of this multi-epitope vaccine candidate suggests that the protein is quite stable and ordered. Furthermore, none of the discontinuous B-cell epitopes of the multi-epitope vaccine protein were from bovine, suggesting that the new molecule would not generate any self-immune response to bovine.

The effective CTL response against the multi-epitope vaccine candidate would be generated only when it is presented to the CD8+ T cells by the antigen presenting cells. The Toll-like receptors (TLRs) which are evolutionarily conserved proteins can sense foreign molecules. They are characterized by an extracellular leucine-rich repeat domain and an intracellular Toll/IL-1 receptor-like (TIR) domain. The TLRs play a fundamental role in the initiation of the immune response to the infectious agents through their recognition of the conserved microbial molecular pattern. Out of all the TLRs, only TLR4 and TLR11 have been shown to recognize the proteins [review (62)]. TLR11 is absent in both human and bovine, thus in our *in silico* analysis we tested the binding ability of multi-epitope vaccine candidate with TLR4 only. We performed homologous modeling of bovine TLR4 using human TLR4 as a template. The binding energy of the designed multi-epitope vaccine candidate and bovine TLR4 was predicted to be −18.3 kcal/mol and the binding sites in the protein were distributed to the various regions of the proteins, suggesting that the bovine TLR4 would effectively recognize the vaccine candidate designed herein. Thus overall in this study, we designed a new vaccine candidate *in silico* containing multiple epitopes of *Theileria* parasites.

## Conclusions

This study shows that the immuno-informatics driven genome-wide screening of vaccine targets for *Theileria* is a highly promising strategy to accelerate vaccine development for this parasite. Based on this strategy, B-cell epitopes and CTLs were mapped from the proteome of the *Theileria* parasite. This study provides a comprehensive analysis of *Theileria* proteins predicted to be better protective immunogens with high conservancy and which would have the potential for eliciting both neutralizing antibodies and T-cell responses. Thus, this study opens new avenues for accelerating vaccine development by providing various potential molecules as novel vaccine candidates and a multi-epitope based vaccine candidate.

## Author contributions

AS conceived and planned the work. AS and PK generated and analyzed data. Both authors shaped the research, analysis and wrote the manuscript.

### Conflict of interest statement

The authors declare that the research was conducted in the absence of any commercial or financial relationships that could be construed as a potential conflict of interest.

## Supplementary material

Figure S1 **(A)** Molecular modeling of antigenic proteins of *Theileria annulata* containing B-cell epitope using Raptor X. The red color represent buried epitopes and blue represent exposed using TMHMM, **(B)** Ramachandran plot analysis of modeled antigenic proteins of *T. annulata* containing B-cell epitope using Raptor X.

Figure S2 Ramachandran plot analysis of bovine MHC-I alleles predicted by Modeler 9.19 showing that all the predicted model are high quality model with more than 90% residue are in core region. **(A)** BoLA-3^*^05101 **(B)** BoLA-1^*^00901 **(C)** BoLA-1^*^00902.

Figure S3 **(A)** Secondary structure of multi-epitope vaccine protein shows 90% residue are helix, 9% residue are coil and no beta sheet are present in the structure **(B)** Conformational B-cell epitope in the multi-epitope vaccine protein predicted by EliPro server. The multi-epitope vaccine protein was represented in red color with ribbon model. There are nine conformational B-cell epitope are present in the final multi-epitope subunit vaccine protein showed in different color with surface model.

Table S1 Annotation of antigenic proteins.

Table S2 Antigenic protein prediction using Vaxijen server with threshold 0.5.

Table S3 B-cell epitope prediction using BCPREDS with specificity 90%.

Table S4 Analysis of epitopes present in cytoplasm, transmembrane and extracellular regions using TMHMM server.

Table S5 T cell epitope prediction using NetCTL with a threshold of 0.98.

Table S6 Identification of MHC-I restriction elements.

Table S7 Identification of CTL epitopes generated in multi-epitope vaccine candidate.

Table S8 Identification of B-cell epitopes generated in multi-epitope vaccine candidate.
