# Supplementary figures and images for: Immuno-informatics Analysis to Identify Novel Vaccine Candidates and Design of a Multi-Epitope Based Vaccine Candidate Against Theileria parasites
Source: Front Immunol. 2018 Oct 15;9:2213. doi: 10.3389/fimmu.2018.02213 (PMC6197074; doi:10.3389/fimmu.2018.02213)

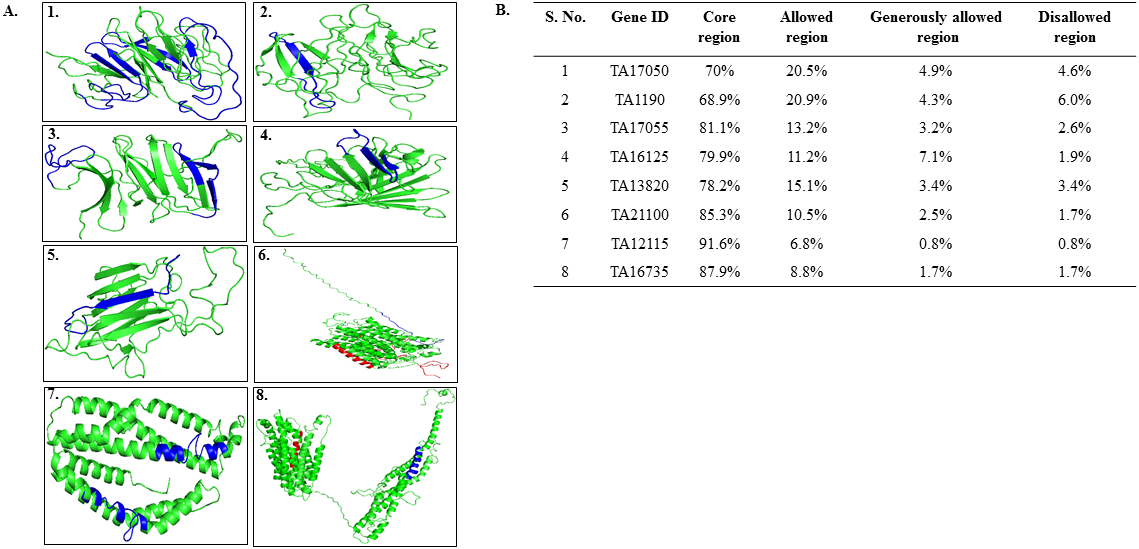

Supplement: Figure S1 — (A) Molecular modeling of antigenic proteins of Theileria annulata containing B-cell epitope using Raptor X. The red color represent buried epitopes and blue represent exposed using TMHMM, (B) Ramachandran plot analysis of modeled antigenic proteins of T. annulata containing B-cell epitope using Raptor X. [file Image_1.TIF]

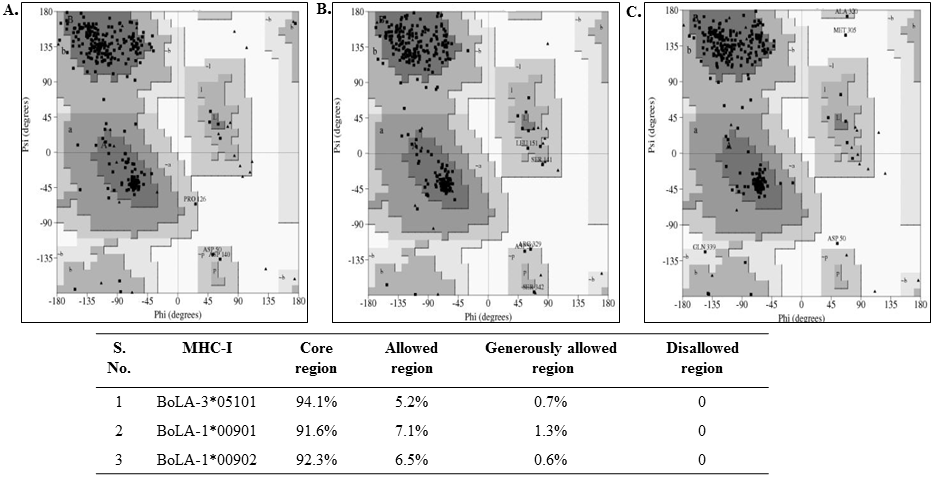

Supplement: Figure S2 — Ramachandran plot analysis of bovine MHC-I alleles predicted by Modeler 9.19 showing that all the predicted model are high quality model with more than 90% residue are in core region. (A) BoLA-3*05101 (B) BoLA-1*00901 (C) BoLA-1*00902. [file Image_2.TIF]

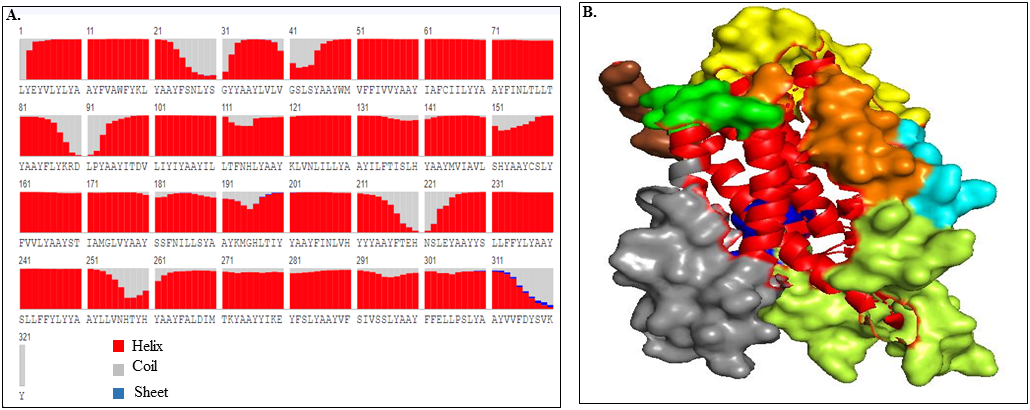

Supplement: Figure S3 — (A) Secondary structure of multi-epitope vaccine protein shows 90% residue are helix, 9% residue are coil and no beta sheet are present in the structure (B) Conformational B-cell epitope in the multi-epitope vaccine protein predicted by EliPro server. The multi-epitope vaccine protein was represented in red color with ribbon model. There are nine conformational B-cell epitope are present in the final multi-epitope subunit vaccine protein showed in different color with surface model. [file Image_3.TIF]
